# Alcohol as an Etiologic Factor in Insanity

**Published:** 1915-12

**Authors:** 


**Alcohol as an Etiologic Factor in Insanity.** The Institution Quarterly, representing the various activities of the State of Illinois, states that of 460 consecutive admissions to one male reception ward, 97 were alcoholic psychoses—21%.

